# Supplementary material for: End Sequence Analysis Toolkit (ESAT) expands the extractable information from single-cell RNA-seq data
Source: Genome Res. 2016 Oct;26(10):1397–410. doi: 10.1101/gr.207902.116 (PMC5052061; doi:10.1101/gr.207902.116)
Supplement: Supplemental Material [file supp_gr.207902.116_Supp_Materials.tar.gz › ESAT-esat_v0.1/doc/html/functions_e.html]

ESAT: Class Members


ESAT


- Main Page
- Related Pages
- Packages
- Classes
- Files

- Class List
- Class Index
- Class Hierarchy
- Class Members

- All
- Functions
- Variables
- Enumerator

- a
- b
- c
- d
- e
- f
- g
- h
- i
- j
- k
- l
- m
- n
- o
- p
- q
- r
- s
- t
- u
- v
- w
- z


All Classes Namespaces Files Functions Variables Enumerator Pages

Here is a list of all class members with links to the classes they belong to:

### - e -

- EciI
  : broad.core.primer3.RestrictionEnzyme
- EcoP15I
  : broad.core.primer3.RestrictionEnzyme
- eig()
  : Jama.Matrix
- EigenvalueDecomposition()
  : Jama.EigenvalueDecomposition
- emitObservation()
  : broad.core.hmm.MarkovState< T >
- emitPath()
  : broad.core.hmm.MarkovModel< T >
- EmpiricalDistribution()
  : broad.core.math.EmpiricalDistribution
- encode()
  : broad.core.multiplealignment.MAFAlignment
  , broad.core.multiplealignment.MultipleAlignment
- encodeAsMatrix()
  : broad.core.multiplealignment.MAFAlignment
  , broad.core.multiplealignment.MultipleAlignment
  , broad.core.siphy.TreeScaler
- encodeSequence()
  : broad.core.sequence.Sequence
- encodeSequenceAsVector()
  : broad.core.sequence.Sequence
- encodeSequenceIgnoreCase()
  : broad.core.sequence.Sequence
- EndInWindowCountScore()
  : umms.core.model.score.EndInWindowCountScore
- ENFORCE\_REFERENCE
  : umms.core.annotation.AnnotationList< T extends Annotation >
- EnrichmentMetric()
  : broad.pda.enrichment.EnrichmentMetric
- ensureNoEmptyBins()
  : broad.core.math.EmpiricalDistribution
- entropy()
  : broad.core.math.Statistics
- enumerateAllKMers()
  : broad.core.primer3.ComputeOriginalScore
- eof()
  : broad.core.annotation.AnnotationHandler
  , broad.core.sequence.FastaHandler
  , broad.pda.datastructures.LocationAwareMatrix
- equalizeTranscriptEnds()
  : broad.pda.annotation.BEDFileParser
- equals()
  : broad.core.datastructures.Pair< T1 >
  , broad.core.motif.SequenceMotif
  , broad.core.sequence.SequenceRegion
  , broad.pda.seq.graph.Path
  , umms.core.alignment.FeatureSequenceAlignment.UnorderedGenePair
  , umms.core.alignment.FragmentAlignment
  , umms.core.alignment.PairedReadAlignment
  , umms.core.alignment.SingleEndAlignment
  , umms.core.annotation.AbstractAnnotation
  , umms.core.annotation.Annotation
  , umms.core.annotation.CompoundInterval
  , umms.core.annotation.SingleInterval
  , umms.core.feature.GeneWindow
  , umms.core.model.score.BasicScanStatisticScore
  , umms.core.model.score.CountScore
  , umms.core.model.score.ScanStatisticScore
  , umms.core.readFilters.SplicedReadFilter
- ESAT()
  : umms.esat.ESAT
- ESATDistanceCalculator()
  : umms.esat.ESATDistanceCalculator
- EstimateOmegaPerExon()
  : broad.core.siphy.tools.conservation.EstimateOmegaPerExon
- euclideanDistance()
  : broad.core.math.Statistics
- evaluate()
  : nextgen.core.readFilters.GenomicSpanFilter
  , nextgen.core.readFilters.PairedAndProperFilter
  , nextgen.core.readFilters.ReadsToReconstructFilter
  , umms.core.annotation.filter.FullyContainedFilter
  , umms.core.annotation.filter.OverlapFilter
  , umms.core.readFilters.MappingQualityFilter
  , umms.core.readFilters.PairedEndFilter
  , umms.core.readFilters.PCRDuplicateFilter
  , umms.core.readFilters.ReadEndsInFilter
  , umms.core.readFilters.ReadStartsInFilter
  , umms.core.readFilters.SameOrientationFilter
  , umms.core.readFilters.SplicedReadFilter
  , umms.core.readFilters.UniqueMappedReadsFilter
- EvolutionaryModel()
  : broad.core.siphy.EvolutionaryModel
- EvolutionaryModelParameters()
  : broad.core.siphy.EvolutionaryModelParameters
- evolve()
  : broad.core.siphy.EvolutionaryModel
- EXCLUDE\_BED
  : broad.pda.seq.rap.DistanceToBED
  , broad.pda.seq.rap.rna.CollectRnaSeqMetrics
- EXCLUDE\_REGION\_FILE
  : broad.pda.seq.rap.rna.CollectRnaSeqMetrics
- excludeByRowNames()
  : broad.core.datastructures.MatrixWithHeaders
- expand()
  : broad.core.sequence.SequenceRegion
  , umms.core.annotation.AbstractAnnotation
  , umms.core.annotation.Annotation
- expandUtrs()
  : broad.pda.gene.GeneWithIsoforms
  , umms.core.annotation.Gene
- expanedUtrs()
  : broad.pda.annotation.BEDFileParser
- EXPRESSION\_PVALUE\_CUTOFF
  : broad.pda.seq.protection.TwoSampleScanSkellamPeakCaller
- expressionByPval
  : broad.pda.seq.protection.WindowScores
- expressionByScanPval
  : broad.pda.seq.clip.SampleData
  , broad.pda.seq.protection.SampleData
- expressionCutoffValue
  : broad.pda.seq.clip.SampleData
  , broad.pda.seq.protection.SampleData
  , broad.pda.seq.protection.WindowScores
- expressionData
  : broad.pda.seq.protection.MultiSampleScanPeakCaller
- ExpressionExperimentInfo()
  : broad.pda.geneexpression.ExpressionExperimentInfo
- extendAnnotation()
  : umms.core.annotation.Gene
- extendPairedEndReads()
  : broad.pda.samtools.PairedEndTDF
- extendSuperArgsForSampleAndChr()
  : broad.pda.seq.protection.BatchedMultiSampleScanPeakCaller
- extractBarcodeFromFastq()
  : broad.pda.seq.fastq.SplitFastqByIndex
- extractGFFRegions()
  : broad.core.multiplealignment.AlignmentExtractor
- Extractor()
  : broad.core.sequence.Extractor
- extractRecords()
  : broad.core.sequence.FastaSequenceIO
- extractRecordsIntoSequenceList()
  : broad.core.sequence.FastaSequenceIO
- extractRecordsWithIDLike()
  : broad.core.sequence.FastaSequenceIO
- extractRecordsWithIDsMatching()
  : broad.core.sequence.FastaSequenceIO
- extractRegion()
  : broad.core.sequence.FastaSequenceIO
  , broad.pda.feature.genome.Chromosome
- extractRegionBasedOnGC()
  : broad.core.sequence.Sequence
  , broad.core.sequence.SequenceRegion
- extractRegions()
  : broad.core.sequence.FastaSequenceIO
  , broad.pda.feature.genome.Chromosome
- ExtractSamplesAndLabel()
  : broad.pda.geneexpression.agilent.ExtractSamplesAndLabel


---

Generated on Fri Aug 15 2014 15:11:16 for ESAT by  

 1.8.7
